# Identification of phases, symmetries and defects through local crystallography

**DOI:** 10.1038/ncomms8801

**Published:** 2015-07-20

**Authors:** Alex Belianinov, Qian He, Mikhail Kravchenko, Stephen Jesse, Albina Borisevich, Sergei V. Kalinin

**Affiliations:** 1 Institute for Functional Imaging of Materials, Oak Ridge National Laboratory, Oak Ridge, Tennessee 37831, USA; 2 The Center for Nanophase Materials Sciences, Oak Ridge National Laboratory, Oak Ridge, Tennessee 37831, USA; 3 Materials Sciences and Technology Division, Oak Ridge National Laboratory, Oak Ridge, Tennessee 37831, USA

## Abstract

Advances in electron and probe microscopies allow 10 pm or higher precision in measurements of atomic positions. This level of fidelity is sufficient to correlate the length (and hence energy) of bonds, as well as bond angles to functional properties of materials. Traditionally, this relied on mapping locally measured parameters to macroscopic variables, for example, average unit cell. This description effectively ignores the information contained in the microscopic degrees of freedom available in a high-resolution image. Here we introduce an approach for local analysis of material structure based on statistical analysis of individual atomic neighbourhoods. Clustering and multivariate algorithms such as principal component analysis explore the connectivity of lattice and bond structure, as well as identify minute structural distortions, thus allowing for chemical description and identification of phases. This analysis lays the framework for building image genomes and structure–property libraries, based on conjoining structural and spectral realms through local atomic behaviour.

## Abstract (web summary)

High-resolution microscopy methods provide a rich source of information, and allow highly precise measurements of atomic coordinates. Here, the authors report a method for quantitative analysis of material structures using multivariate statistical analysis to identify and distinguish various phases, defects and symmetries.

## Graphical abstract

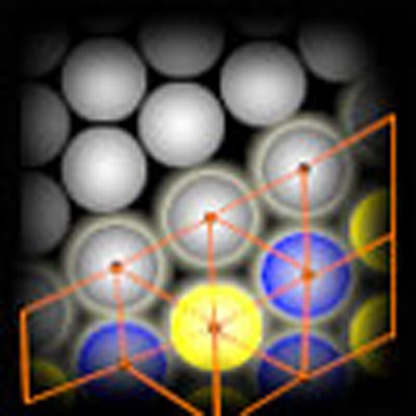

The introduction of scattering techniques in the beginning of twentieth century by the Braggs has paved the way for probing the structure of matter on the atomic scales^1^. Early milestones include structure identification of simple crystalline substances as well as DNA, with recent advances encompassing small-angle scattering, radial distribution function analysis, inelastic scattering methods and surface diffraction and ptychography^2^. Despite the broad variety of scattering techniques, the basic principle—analysis of the structure factor—or equivalently a pair correlation function averaged over the probing volume remained invariant since the early days of the Braggs team. Furthermore, operating in the reciprocal space, natural to the scattering-based techniques, forged the way many generations of condensed matter scientists think. In fact, working in *k*-space to explore elementary excitations and normal modes can be considered a classical approach to physics. Typically, these surface or lattice descriptions are based on the periodicity of the system in real space and are intrinsically linked to the underlying symmetry. Unsurprisingly, cases where such description fails, including quasicrystals, nanoscale phase separation in strongly correlated oxides^3^,^4^, morphotropic materials and relaxors^5^,^6^,^7^; remain a topic of much scientific excitement. For all these cases, the knowledge of the structure factor alone is insufficient to reconstruct the lattice of the material.

The progress in high-resolution, real space imaging techniques such as (scanning) transmission electron microscopy (STEM)^8^,^9^,^10^ and scanning tunnelling microscopy (STM)^11^,^12^ have allowed direct imaging of atomic columns (STEM) and surface atomic structures (STM). From the beginning of twenty-first century, the resolution (more specifically, information limit) of these methods has steadily risen to a level where minute displacement of atoms, from idealized high symmetry positions, can be visualized and quantified with high veracity. The examples in the field of aberration corrected (S)TEM include direct imaging of ferroelectric polarization^13^,^14^,^15^,^16^, octahedral tilts^17^,^18^ and chemical expansion strains^19^. Another example is high-resolution STM, allowing direct visualization of octahedral tilts^20^, surface strains^21^, complex structural reconstructions^22^ and Jahn–Teller distortion fields^23^. In this manner, not only atomic structure but also subatomic order parameter fields can be visualized.

Typically, such image-based analyses are based on implicit, *a priori*, assumptions of the macroscopic symmetry of the system. These approaches fail when multiple crystallographic phases and/or extended defects are present. Finding atoms without a reference to a global lattice is a general particle search problem, a well explored area with multiple available algorithms. That said, adaptation of these algorithms to an atom search is non-trivial, especially when multiple atom types are present. Other approaches circumvent atom finding by analysis of image segments that contain features of interest to extract relevant information^24^,^25^. However, contrast-based image analysis methods are prone to error propagation, especially in the case of lower-quality images, significant computational time cost for large images and multiple image arrays, and rather extensive user involvement and expertise. Notably, steps have been taken to ameliorate these complications through the use of correcting algorithms including parameters such as orientation of the detector and environmental distortions^26^. These image processing techniques are quite powerful and ensure maximal data veracity before the analysis is initiated. Conjoined with such a powerful and extensive suite of image processing software, contrast-based methods have achieved impressive results that extend into the realm of three-dimensional reconstruction and internal structure mapping^27^.

Nevertheless, once all atoms are found, a bigger challenge arises: without the global lattice as an intuitive vehicle for interpretation of the local structural data, we need to find completely new ways to categorize, analyse and interpret. Here we aim to explore whether a universal physical description of the system, including local and global symmetries, phases, and topological defects, can be built up only from the local information, obviating the overall lattice structure^28^. We propose an approach based on the multivariate statistical analysis of the coordination spheres of individual atoms, made up by an array of values that represent a variety of metrics between an atom and its nearest or next nearest neighbours, to reveal preferential structures and symmetries. We test this approach on a mixed-phase system with a variety of nearest neighbour environments and show how a framework for interpretation of this new type of structural data can be developed.

## Results

### Algorithm workflow

We define the chemical neighbourhood of the atom via the number and identity of the nearest neighbours. The types of atoms with statistically different chemical neighbourhoods and their spatial distribution define chemical composition, that is, phases. If the chemical neighbourhoods are related by point symmetry operations, such as rotation or mirror symmetry, this defines physical ferroic variants. Note that classical definitions of phase and ferroic variants further rely on the presence of translation symmetries, and below we demonstrate the development of a local picture and discuss possible pathways for global description.

**Figure 1:**
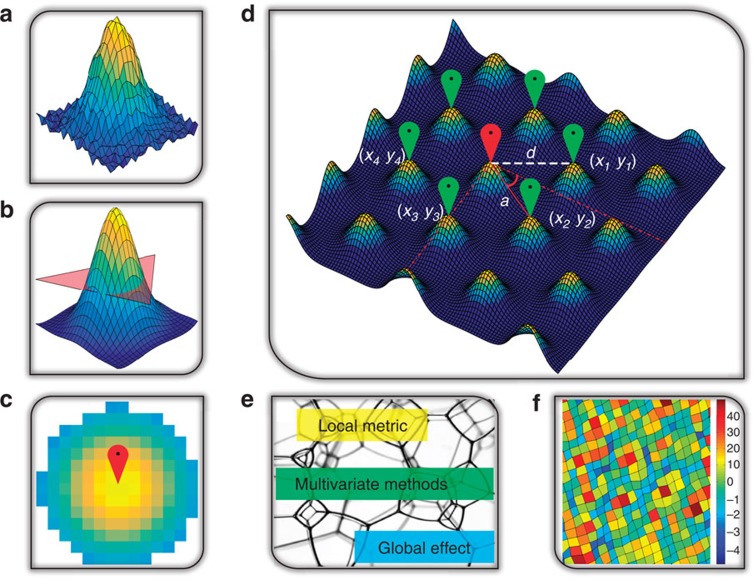
Operational workflow of atom finding and subsequent analysis. (**a**) A representative atom (target) extracted from an image or supplied by the user. (**b**) Threshold the atom after correlation analysis. (**c**) Finding the centre of the atom in a binary image after a threshold in **b**. (**d**) A central atom (labelled no. 1) and six neighbours with distance and angle metric assignments shown. (**e**) Artistic representation of converting image data into vector data. At this step atomic centres from and their neighbourhoods are compiled into a single array on which multivariate analysis is performed. (**f**) Visualization of the multivariate analysis results.

**Figure 2:**
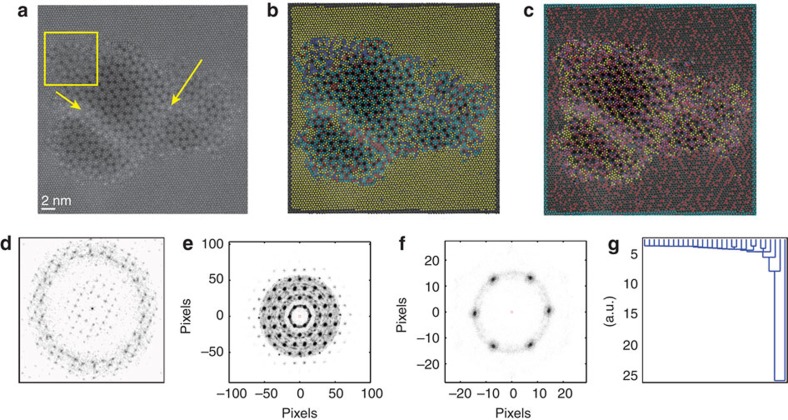
Two-phase Mo–V–Te–Ta oxide. (**a**) M1 and M2 mixed-phase STEM image. (**b**) *k*-means clustering results for six neighbours, sorted by distance metric. (**c**) *k*-means clustering results for six neighbours, sorted by angle metric. (**d**) FFT of image in **a**. (**e**) Fifty member neighbourhood of the image in **a**. (**f**) Six member neighbourhood of the image in **a**. (**g**) Dendrogram for the six neighbour, sorted by distance metric, with the *y* axis signifying the cluster separation in the hierarchical tree.

The schematic of our near-atomic neighbourhood-based approach is illustrated in Fig. 1. Before the image enters the analysis workflow, it is lightly preprocessed to remove jitter noise and normalize contrast. As a first step of analysis, we identify all atoms in the image. Classically, this is achieved by overlaying a coarse ideal atomic lattice based on periodicity or Fast Fourier Transform (FFT) filtering and then relaxing the ideal lattice until an appropriate level of fit is found. In the case shown in Fig. 2a, this global approach is not applicable due to the lack of long-range periodicity across the image, and hence a local approach is used. The local approach works by first approximating a typical atomic shape (Fig. 1a). This is performed either manually or automatically by identifying a set, or a representative member in the image. Following the target identification, we perform a correlation of the target across the entire image over each pixel, by sliding the target window over the image. This produces a correlation map where locations of the target are strongly highlighted and areas that do not match the target are suppressed. The correlated surface is then thresholded to remove any small artefacts, resulting in a binary image where all areas are zero, except those that correlate strongly with the target (Fig. 1b). The binary image is processed further using a size histogram, to remove any point artefacts; we also found this procedure well suited for images with varying intensity backgrounds, as size of the object will vary with the background. The centroids of the final, disconnected objects in the image are identified through standard image processing functions available in Matlab Image Processing Toolbox (Fig. 1c). With the centroids identified, position refinement and the multivariate analysis steps can be launched (Fig. 1d–f).

As a model system, we have chosen mixed oxide Mo–V–M–O (M=Nb, Ta, Te and/or Sb), which is currently a promising catalyst for many industrially important reactions, such as propane (amm)oxidation^29^. In this system, the following phases are noted for their catalytic performance: the orthorhombic M1 phase with a space group Pba2 and a pseudo-hexagonal M2 phase with a space group Pmm2. The M1 phase, containing Mo_6_O_21_-type pentagonal units and doublet heptagonal channels, is the main active phase for paraffin activation, while the M2 phase, containing only hexagonal channels, has a possible synergistic effect when used together with the M1 by improving the reaction selectivity^30^. We recently discovered that these two phases can actually form coherent interfaces and intergrowths, suggesting new directions for catalyst improvement^31^. STEM images were taken with relatively fast scanning speed (that is, 1 μs per pixel). The displayed image were the summation of multiple (that is, 30) fast sequentially scanned images aligned via cross-correlation. In this way, the image artefacts^26^ due to scan noise, possible beam damage and drift can be minimized while the signal-to-noise ratio is maintained. A representative image showing the coexistence of M1 and M2 phases is shown in Fig. 2a. Note that the image contains multiple clearly visible regions with different crystalline ordering separated by a boundary (emphasized by yellow arrows) that contains lattice elements from both regions (highlighted by a yellow rectangle).

Using defined atomic shape as a template at the first step of the processing flow offers a distinct advantage in being able to differentiate sublattices as well as tilts or other contrast-based features. The correlation step is sensitive to small details of the supplied shape as well as its size, maximizing selectivity of finding the member of interest. Finally, flooding and histogram binning allow any remaining small features, such as edge artefacts and intensity imbalances, to be removed. Once atom positions are identified, the centres are refined with sub-pixel precision. Since the approximate centres are already known from the centroid identification, they are used as a seed fit for a two-dimensional (2D) Gaussian function that determines the best fit in the radial area of an atom. The maximum of the smooth function is extracted, resulting in an accurate measurement of the centre point of an atom with a higher precision than native resolution.

### Implemented statistical framework

This analysis yields absolute positions of each atom in an image, as well as local descriptors such as column intensity and peak width determined at the refinement stage. For the task of nearest neighbour environment identification, we are only using atomic positions. For each atom, *i*, in the image, we construct a near-coordination sphere as an array **N**_*i*_=([*x*_1_, *y*_1_]..., [*x*_*j*_, *y*_*j*_]), where (*x*_*j*_, *y*_*j*_) is the position coordinate of the *j*th nearest neighbour. The number of nearest neighbours, or the search radius, can be defined separately and are chosen depending on the analysis. In the simplest case, neighbours are chosen based on dominant symmetry, for example, 6 for hexagonal lattice or 4/8 for cubic lattice. When the number of defined neighbours exceeds the available nearest neighbours, the next nearest neighbours are included. In the case when the search radius is used, the returned number of neighbours varies for each atom, due to vacancies (in the case of STM images), image edges or different coordination numbers.

At the first step, we explore statistical properties of neighbour distributions. Shown in Fig. 2e,f are statistical distributions for defined neighbourhoods of 50 and 6 neighbours, respectively. For a large number of neighbours, the derived distribution effectively represents the 2D pair correlation function illustrating the global periodicity in the image. For a smaller number of components, the average structure of the nearer chemical neighbourhood is revealed. In both cases, the maxima in the distribution correspond to preferential inter atomic distances in a hexagonal lattice. However, in the Fig. 2f, additional intermediate points are observed that do not fall into the hexagonal maxima. This is due to the local environments in the image that do not follow the same symmetry. Note that while six nearest neighbours were used for the analysis, this approach can be extended for more remote neighbours and incorporate multiple sublattices. In addition, the central atom to neighbour relationship can be further explored by classification of the members in the coordination sphere by arranging them by length to the centre or angle of the bonds and so on. That is, using the **N**_*i*_ vector as a descriptor for a particular behaviour of interest, as illustrated in Fig. 2b,c.

Once the set of **N**_*i*_ vectors is assembled, the data object can be analysed as a multispectral data set via multivariate statistical methods. To identify the chemical structure of material, we perform clustering analysis of local neighbourhoods, effectively establishing the types of chemical environments. Here we utilize a *k*-means clustering algorithm to divide *i* points (or their corresponding **N**_*i*_ vectors) into *K* clusters so that the within cluster sum of squares is minimized (Equation 1).


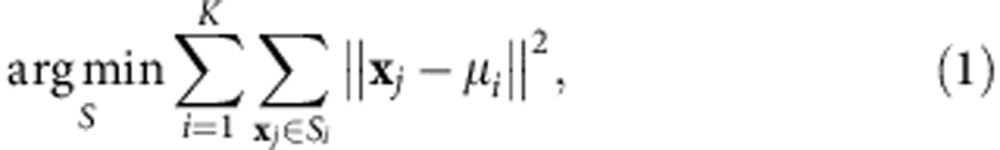


here *μ*_*i*_ is the mean of all points in *S*_*i*_. We use the square Euclidean distance with each centroid being the component wise median of the points in a given cluster. The clustering is performed as a function of number of clusters, *K*, and the quality of separation, which can be represented as a dendrogram (explained in detail below), allowing a range of optimal number of clusters to be determined. Thus, determined clusters define the groups of atoms with specific chemical neighbourhoods that can be further positioned in real space and corresponding configurations can be explored through direct visualization, classical correlation function and Fourier transform methods.

**Figure 3:**
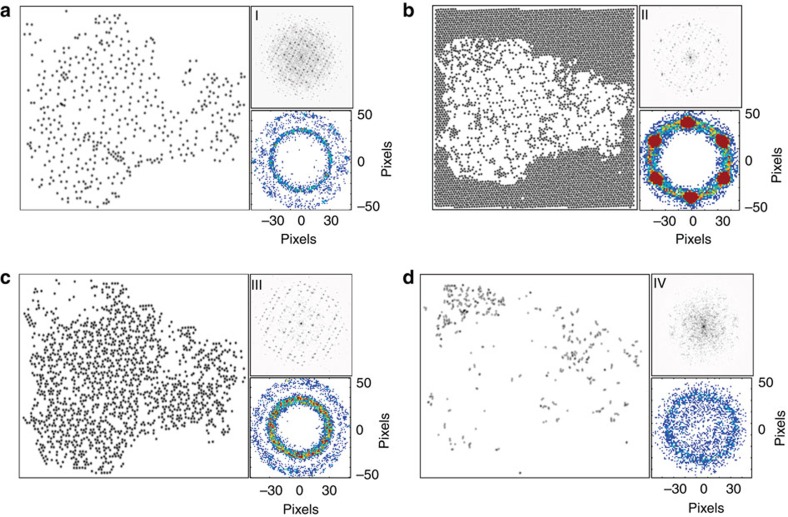
Individual *k*-means clusters for image in Fig. 2, distance metric. (**a**) Cluster 1 spatial distribution with (I) FFT of the distribution and a 2D histogram of neighbours of atoms in the cluster. (**b**) Cluster 2 spatial distribution with (II) FFT of the distribution and a 2D histogram of neighbours of atoms in the cluster. (**c**) Cluster 3 spatial distribution with (III) FFT of the distribution and a 2D histogram of neighbours of atoms in the cluster. (**d**) Cluster 4 spatial distribution with (IV) FFT of the distribution and a 2D histogram of neighbours of atoms in the cluster.

Shown in Fig. 2g is a dendrogram for the image shown in Fig. 2a based on a classification of the distance to the central atom in a six neighbour case. A dendrogram plot illustrates hierarchical cluster arrangement in a top-down approach, where all observations are grouped into a single cluster initially and are recursively separated down the hierarchy. This is achieved by establishing a distance metric between observations and linkage criteria used to find the dissimilarity of clusters as a function of pairwise distances. It then follows that vertical axis in the dendrogram plot represents the distance between the two data points being connected (by whatever metric of choice), and the largest drops indicate major changes in data organization. On the basis of dendrogram for Fig. 2g, the strongest cluster separation occurs for two, three and four clusters with more than four clusters being the limit of significant optimization gain. We have found that a large percentage of total clusters identify strong outliers, that is, points found at the edges of the image, which while rigorously correct does not add to the understanding of the material being imaged. Therefore, we chose to omit the atoms that lie on the image boundary as centre atoms; however, their positions are still utilized as neighbours for the atoms further inward in the image. The results of four cluster separation based on the distance metric are shown in Fig. 2b, and for four clusters with an angle metric in Fig. 2c. Rotation is accounted for by always placing the first neighbour atom in the same location relative to the centre and filling the rest in a clockwise manner. Figure 3a–d shows where the atoms from each of the clusters are located on the initial image, with an accompanying FFT (Fig. 3(I–IV)) of the cluster points illustrating the symmetry of their relative distribution, as well as a 2D histogram of the nearest neighbour environment for atoms within the cluster.

Note that the analysis clearly distinguishes different areas of the image based on the similarity of chemical neighbourhoods of their constituent atoms. The coordination environments in Fig. 3a,c,d exist only within the central grain. The component in Fig. 3d forms a clearly visible region at the boundary between the grain and the outer matrix, characterized by least long-range order as is evident from the FFT in Fig. 3 IV; the Fig. 3b defines the matrix. Broadly, components in Fig. 3a–c have clear long-range periodicity in space, corresponding to specific sites within the unit cells of the respective phases. Note that in this case, the spatial periodicity of the individual clusters does not follow from, or contribute to classification, since the latter relies purely on the properties of nearest neighbourhood and does not contain any information regarding the long-range order in the system; rather, we are using FFT as a post processing approach allowing us to differentiate between periodic and non-periodic classes in the initial image.

The relative distribution of the clusters can also be viewed in the form of the colour map as shown in Fig. 2b,c. Examination of the original image (Fig. 2a) and the image with overlaid cluster information (Fig. 2b,c) side by side makes apparent several characteristic patterns. First, the regions of single-phase M2 matrix, several subgrains within the central M1 grain and clearly visible amorphous boundaries separating these regions are distinguished. Second, a region emerges that is omprised of closely located atoms with similar local environments not found in either M1 or M2 phase, which can be tentatively associated with the emergence of a distinct third-phase region. While the first conclusion is also apparent from the visual examination of the initial image, the second one is not, demonstrating the advantages of the statistical analysis of the local neighbourhoods for analysing internal phase composition and structure of partially ordered phases in real space.

**Figure 4:**
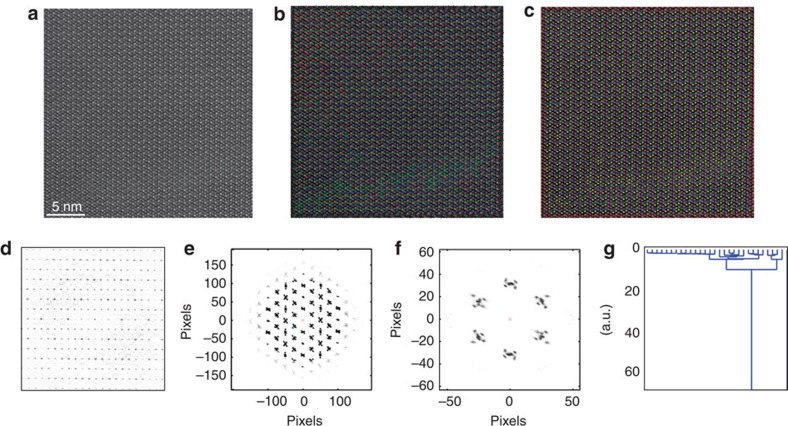
Single M2 phase Mo–V–Te oxide. (**a**) M2 phase STEM image. (**b**) *k*-means clustering results for six neighbours, sorted by distance metric. (**c**) k-means clustering results for 6 neighbours, sorted by angle metric. (**d**) FFT of image in **a**. (**e**) Fifty member neighbourhood of the image in **a**. (**f**) Six member neighbourhood of the image in **a**. (**g**) Dendrogram for the six neighbour, sorted by angles metric, with the *y* axis signifying the cluster separation in the hierarchical tree.

We further extend the multivariate approach to explore minute deviations of the internal structure in a single-phase region. Shown in Fig. 4 is a STEM image of a crystalline region of the M2 phase. The corresponding Fourier transform and nearest neighbour distributions for 50 neighbours and 6 neighbours are shown in Fig. 4d–f, respectively. Note the high degree of crystallinity in the material as reflected in the FFT. Interestingly, the neighbourhood histogram shows the internal structure with peaks having x, linear, and dot-like shapes as seen in more detail in Fig. 4e,f. This histogram is a clear indication that M2 phase can be viewed as a simpler hexagonal structure with several small distortions that vary in a periodic fashion from one primitive cell to another, forming a superstructure. Therefore, the dot-like spots delineate the unit cell for the superstructure (a multiple of the primitive cell), and the distorted spots carry information about the symmetry of the distortions on specific sites within this larger unit cell. The corresponding length and angle *k*-means clustered images are shown in Fig. 4b,c with the individual clusters shown in the Supplementary Fig. 1. Notice the clear delineation of the sites of different symmetry within the phase, as well as a clearly visible antiphase boundary, which is not at all apparent in the original image and hardly evident from the raw data in Fig. 4a.

The presence of the fine structure of the neighbour distribution shown in Fig. 4f suggests the presence of minuscule distortions from ideal symmetry of the primitive cell giving rise to the M2 crystalline structure. To visualize these physical behaviours, we analyse these distortions using the principal component analysis (PCA) of the neighbourhood vector to separate statistically significant deformation of the nearest neighbourhood. PCA^32^,^33^,^34^,^35^,^36^ is used to convert **N** observations into a superposition of orthogonal, linearly uncorrelated eigenvectors *w*_*j*_ shown in equation (2).


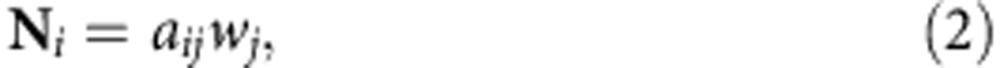


where *a*_*ij*_ are expansion coefficients (PCA loadings). The eigenvectors *w*_*j*_ and the corresponding eigenvalues *λ*_*k*_ are found from the singular value decomposition of the covariance matrix, **C**=**NN**^*T*^, where **N** is the matrix of all experimental data points *N*_*ij*_. That is, the rows of **N** correspond to individual atoms, and columns correspond to *x* and *y* components of radius vectors to nearest neighbours. The eigenvectors *w*_*j*_ are orthogonal and are arranged such that corresponding eigenvalues are placed in descending order, *λ*_1_>*λ*_2_>.... by variance.

**Figure 5:**
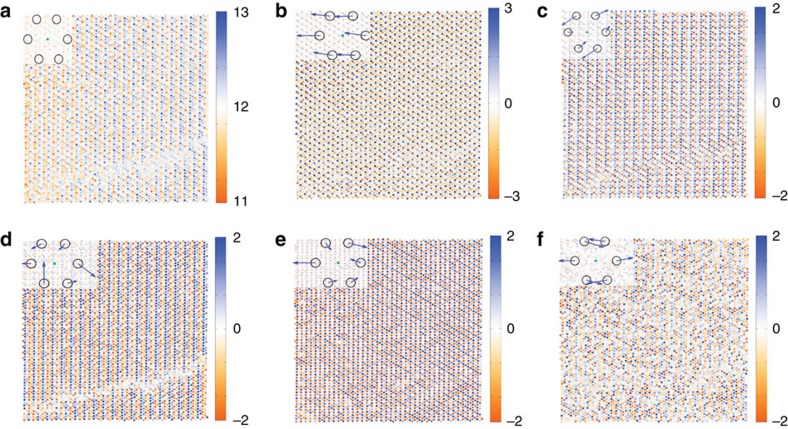
PCA loadings and vectors for the image in Fig. 4a. (**a**) First eigenvector, in the upper left, and a corresponding loading map. (**b**) Second eigenvector, in the upper left, and a corresponding loading map. (**c**) Third eigenvector, in the upper left, and a corresponding loading map. (**d**) Fourth eigenvector, in the upper left, and a corresponding loading map. (**e**) Fifth eigenvector, in the upper left, and a corresponding loading map. (**f**) Sixth eigenvector, in the upper left, and a corresponding loading map.

Shown in Fig. 5a–f are eigenvectors (represented as deviations from the average 6 neighbour shape, located in the upper left corner) and their corresponding loading maps. The scree plot is shown in Supplementary Fig. 2. The first eigenvector is the average, and the loading map shows the residual intensity of all six components. To visualize the higher eigenmodes, we plot them as deformation of the average, represented as vectors of deformation from the ideal lattice positions of the neighbours. We further note that these statistical normal modes do not have well defined physical meaning. Practically, they reveal spatial frequencies present in the image from which symmetry can be inferred, allowing the interpreter to ascribe a likely physical interpretation to some earlier PCA components. For example, the second eigenmode corresponds to the uniform shift of the coordinate sphere along one of the principal directions of the primitive hexagonal cell. In this case, the displacement of the entire nearest neighbour sphere is clearly equivalent to the displacement of the central atom in the opposite direction, a polar distortion. In a more general case, the vector sum of the shifts of the nearest neighbour atoms will determine whether the collective distortion being considered is polar or non-polar in nature (non-zero vector sum versus zero vector sum, respectively). The loading map reflects the previously reported antipolar structure characteristic of the M2 phase; interestingly, the antiphase boundary is not apparent on the loading map, suggesting that the antipolar structure is not altered by the presence of the boundary. The third eigenvector corresponds to a symmetric deformation of the neighbourhood similar to a shear mode. The corresponding map is almost uniform, but close examination shows that the contrast of the map exhibits the shift associated with the antiphase boundary, even though no contrast alteration is associated with the boundary itself. The fourth, fifth and sixth components are more difficult to interpret, as the distortions appear to be very complex. The fifth component is somewhat reminiscent of the rotational transformation of the third. Interestingly, the loading maps associated with the third and forth components show very clear contrast at the antiphase boundary, suggesting that these distortions might be characteristic of the frustrated environment of that defect. In contrast, the loading map for the fifth component, similarly to the second, shows no contrast shift at the antiphase boundary. Information of this type could be tremendously useful when determining polar character of different extended defects.

## Discussion

We have implemented a locality-based analysis of complex materials from high veracity atomically resolved images to explore chemistry and physics at the nanoscale, grounded in the analysis of atomic neighbourhoods. Unlike the classical, symmetry-based descriptions, our approach utilizes local bond characteristics including the structure of the coordination sphere and bonding type. For materials with a significantly varying chemical neighbourhood, this analysis allows identification of the uniform phase regions, as well as clear delineation of unknown phases and structural defects. The Fourier analysis of individual cluster components allows associated symmetries to be revealed.

In the single-phase region, clustering analysis allows decomposition of the system into the elementary sublattices. In this case, additional opportunities are opened by the PCA of local neighbourhoods, defining the statistical normal modes of the system. Again, this statistical description illustrates the predominant statistically significant distortions and ranks them in terms of relevant prevalence.

In general, we believe that this approach paves the way for full information recovery in high-resolution imaging such as electron and scanning probe microscopies, as well as allows for classification and automatic identification of materials. Subsequent effort will be aimed at development of the identification of the repeated statistically defined units based on graph partitioning of underlying lattice, creating a basis for development of image genomes and further development of structure–property correlative libraries based on STEM–EELS and STM–STS data.

## Methods

### Sample

The Mo–V–M oxides were prepared by hydrothermal synthesis or slurry evaporation as previously reported^37^,^38^. Ammonium paramolybdate, telluric acid, antimony trioxide, vanadium (IV) sulfate, niobium (V) oxalate hexahydrate and tantalum (V) ethoxide were used as precursors. All operations, preparation and stirring of the solution, were performed at 353 K except Sb system at 373 K. The slurry was introduced into the Teflon inner tube of a stainless steel autoclave. In the case of slurry evaporation, this slurry was dried overnight in the oven at 383 K. The autoclave was sealed and heated at 448 K for 48 h. After hydrothermal synthesis, the dark blue powder obtained was washed, filtered with distilled water (200 ml) and dried at 353 K for overnight. Then, the dried powder catalyst was calcined under ultrahigh purified nitrogen flow (50 ml min^−1^) at 873 K for 2 h before use. In the case of catalyst containing Sb, the obtained solid was preheated in furnace with air at 573 K for 4 h before calcined under ultrahigh purified nitrogen flow at 873 K for 2 h. The nominal compositions (molar ratio) of the catalysts involved in the work are as follows: Mo–V–Te–Ta oxide shown in Fig. 2, Mo:V:Te:Ta=1:0.3:0.17:0.12; Mo–V–Te oxide shown in Fig. 4, Mo:V:Te=1:0.75:0.75.

### Electron microscopy imaging

To make specimen for electron microscopy, the catalyst sample was embedded in a resin, and sectioned by microtome as ∼50-nm slices^39^. These specimens were introduced into a holey-carbon-coated Cu grid. The HAADF-STEM imaging was performed on UltraSTEM 200 (operated at 200 kV) in Oak Ridge National Laboratory. The inner angle of the High-Angle Annular Dark-Field (HAADF) detector is around 63 mrad. To minimize the beam damage and specimen drift, the images used for analysis were the sum images of 20–30 fast scanned frames (1 μs per pixel and ∼20 pA probe current) stacked with cross-correlation algorithm. Gatan Digitalmicrograph was used for image acquisition, and all the images are 32 bit in depth. No further image processing was performed before PCA, Independent Component Analysis (ICA) and *k*-mean clustering analysis. The pixel size for the original image in Fig. 2 is 0.27 Å and in Fig. 4 is 0.13 Å.

## Additional information

**How to cite this article**: Belianinov, A. *et al.* Identification of phases, symmetries and defects through local crystallography. *Nat. Commun.* 6:7801 doi: 10.1038/ncomms8801 (2015).

## Supplementary Material

Supplementary Information Supplementary Figures 1-2.
