# Supplementary material for: Identification of phases, symmetries and defects through local crystallography
Source: Nat Commun. 2015 Jul 20;6:7801. doi: 10.1038/ncomms8801 (PMC4518243; doi:10.1038/ncomms8801)
Supplement: Supplementary Information — Supplementary Figures 1-2. [file ncomms8801-s1.pdf]

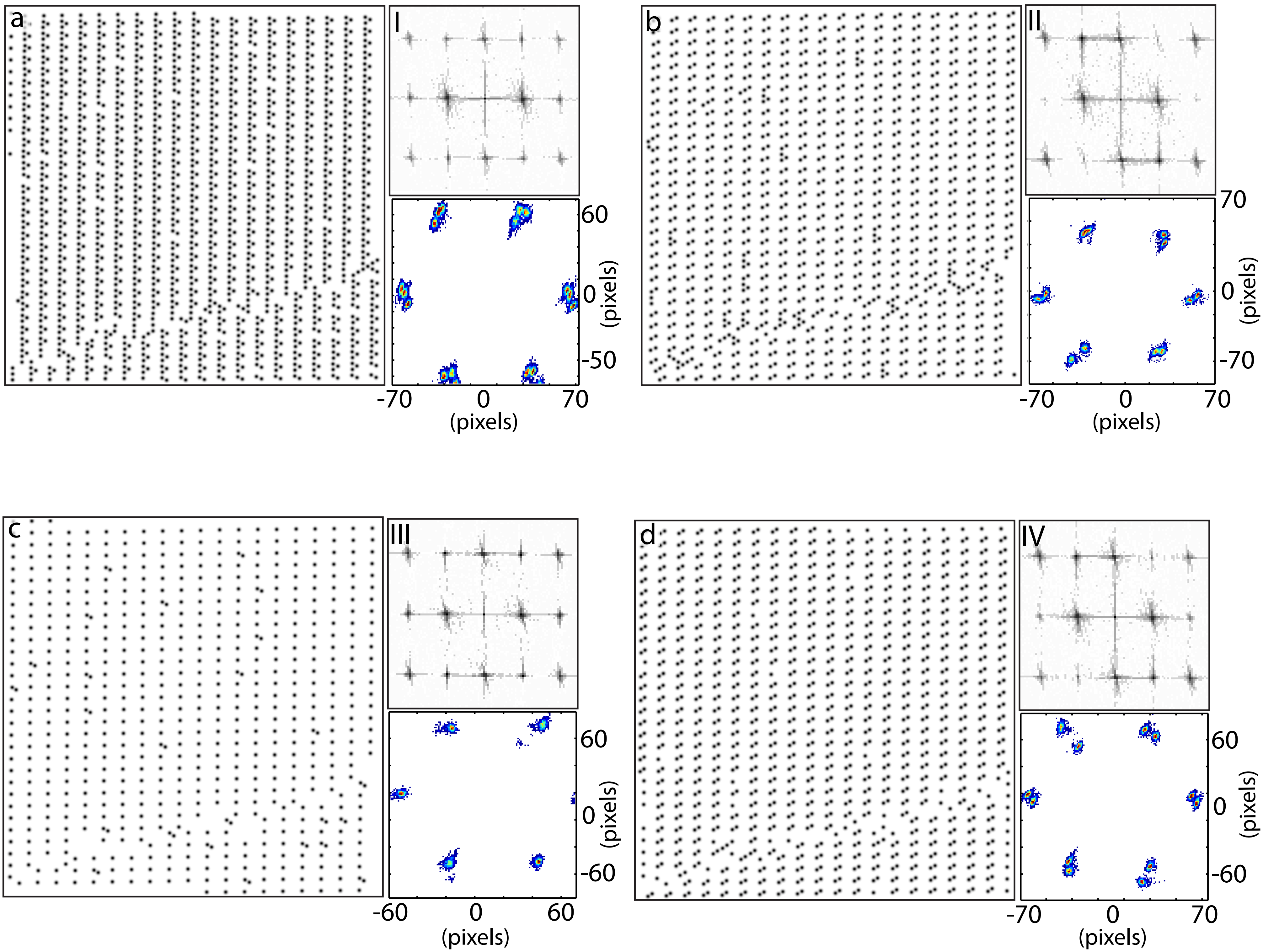
a
I
60
0
-50
(pixels)
-70
0
70
(pixels)
b
II
70
0
-70
(pixels)
-70
0
70
(pixels)
c
III
60
0
-60
(pixels)
-60
0
60
(pixels)
d
IV
60
0
-60
(pixels)
-70
0
70
(pixels)

Supplementary Figure 1. Individual k-means clusters for image in Figure 4, angles metric (a) Cluster 1 spatial distribution with (I) FFT of the distribution and a 2D histogram of neighbors of atoms in the cluster. (b) Cluster 2 spatial distribution with (I) FFT of the distribution and a 2D histogram of neighbors of atoms in the cluster. (c) Cluster 3 spatial distribution with (I) FFT of the distribution and a 2D histogram of neighbors of atoms in the cluster. (d) Cluster 4 spatial distribution with (I) FFT of the distribution and a 2D histogram of neighbors of atoms in the cluster.

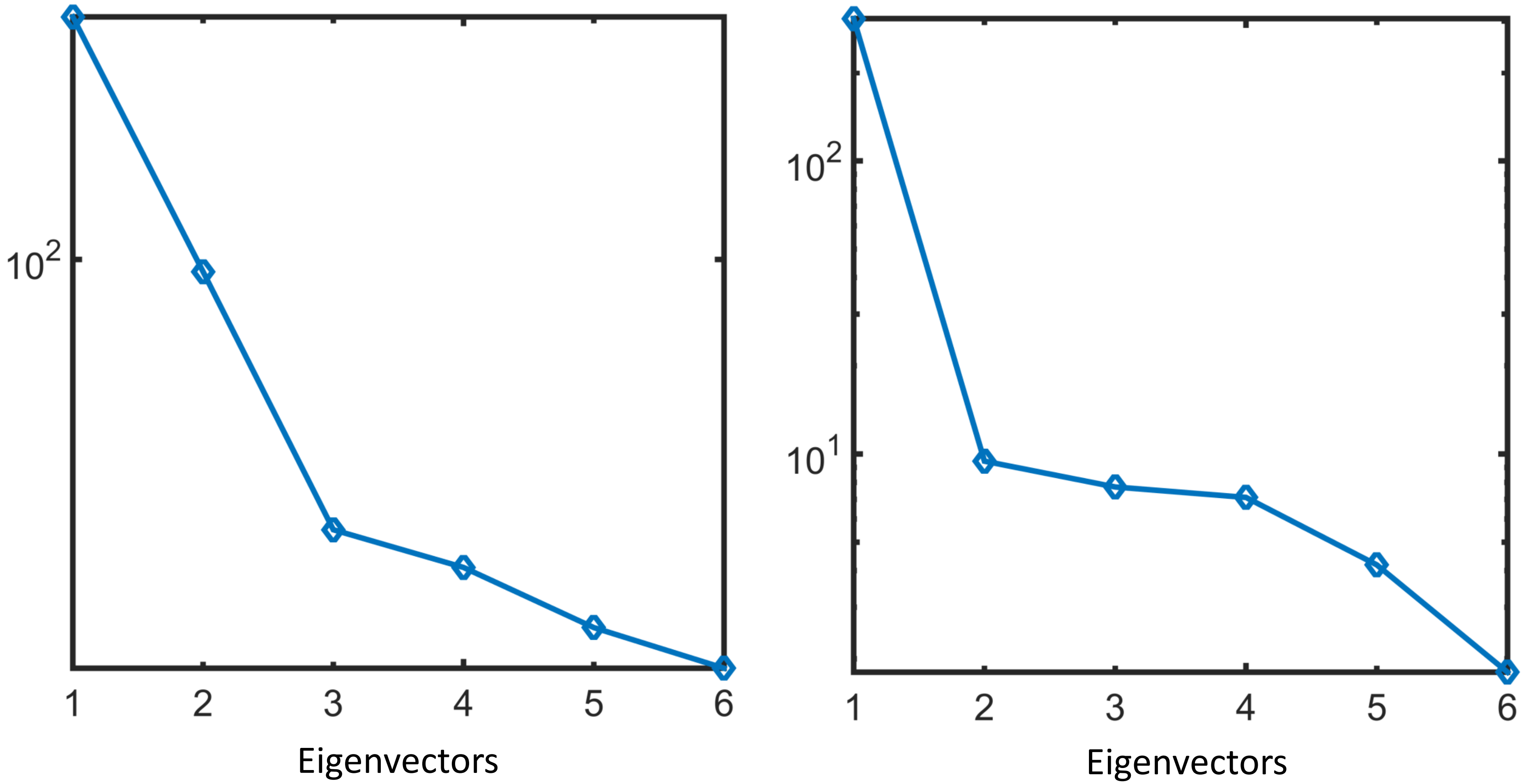

Supplementary Figure 2. Semi-log Scree plots for the PCA analysis of a 6 member neighborhood (a) Scree Results for the raw image shown in Figure 2(a) of the main text. (b) Scree Results for the PCA result data shown in Figure 5(a-f).
